# New Antibacterial and Antioxidant Chitin Derivatives: Ultrasonic Preparation and Biological Effects

**DOI:** 10.3390/polym16172509

**Published:** 2024-09-03

**Authors:** Anton R. Egorov, Omar M. Khubiev, Roman A. Golubev, Daria I. Semenkova, Andrey A. Nikolaev, Abel M. Maharramov, Gunay Z. Mammadova, Wanjun Liu, Alexander G. Tskhovrebov, Andreii S. Kritchenkov

**Affiliations:** 1 Department of Human Ecology and Bioelementology, RUDN University, Miklukho-Maklaya St. 6, 117198 Moscow, Russia; sab.icex@mail.ru (A.R.E.); asdfdss.asdasf@yandex.ru (R.A.G.); darya.semenkova02@mail.ru (D.I.S.); andreynikolaev2001@yandex.ru (A.A.N.); alexander.tskhovrebov@gmail.com (A.G.T.); 2 Metal Physics Laboratory, Institute of Technical Acoustics NAS of Belarus, General Lyudnikov Ave. 13, 210009 Vitebsk, Belarus; 3 Organic Chemistry Department, Baku State University, Z. Khalilov Street, 23, 1148 Baku, Azerbaijan; amaharramov@bsu.edu.az (A.M.M.); gunka479@mail.ru (G.Z.M.); 4 Key Laboratory of Textile Science & Technology, Ministry of Education, College of Textiles, Donghua University, Shanghai 201620, China; wjliuok@dhu.edu.cn; 5 Engineering Research Center of Technical Textiles, Ministry of Education, College of Textiles, Donghua University, Shanghai 201620, China; 6 Shanghai Engineering Research Center of Nano-Biomaterials and Regenerative Medicine, Donghua University, Shanghai 201620, China

**Keywords:** chitin, phenol-ene coupling, ultrasonic reaction, antibacterial activity

## Abstract

This work focuses on the first use of ultrasonic phenol-ene coupling as a polymer analogous transformation. The ultrasonic reaction was introduced into chitin chemistry, resulting in the fast and convenient preparation of new water-soluble cationic chitin derivatives. Since water-soluble derivatives of fully deacetylated chitin are poorly described in the literature, the synthesis of each new type of these derivatives is a significant event in polysaccharide chemistry. Polycations, or cationic polymers, are of particular interest as antibacterial agents. Consequently, the resulting polymers were tested for their antibacterial activity and toxicity. We found that the highly substituted polymer of medium molecular weight exhibited the most pronounced in vitro antibacterial effect. We prepared nanoparticles using the ionic gelation technique. The most effective in vitro antibacterial chitin-based systems were tested in vivo in rats. These tests demonstrated outstanding antibacterial effects combined with an absence of toxicity. Additionally, we found that the resulting polymers, unlike their nanoparticle counterparts, also exhibited strong antioxidant effects. In summary, we demonstrated the effectiveness of ultrasound in polymer chemistry and highlighted the importance of the sonochemical approach in the chemical modification of polysaccharides. This approach enables the synthesis of derivatives with improved physicochemical and biological properties.

## 1. Introduction

Click chemistry has profoundly enhanced organic synthesis with its elegance and efficiency, emerging as a robust tool for synthesizing complex molecules, molecular assemblies, and machines. It has also pioneered effective pathways for bioconjugation, significantly advancing various branches of chemistry [1,2,3,4]. The transformative impact of click chemistry on chemical synthesis and the mindset of synthetic chemists has been extensively analyzed in recent reviews and publications [5,6,7].

Moreover, click reactions have revitalized the chemistry of chitosan, a promising biocompatible and biodegradable natural polymer [8]. Previously constrained by the limitations of classical organic reactions and the insolubility of chitosan in organic solvents (typically soluble only in acidic aqueous environments) [9], click chemistry now enables efficient and mild polymer-analogous transformations of chitosan [10]. This breakthrough has led to the development of novel chitosan-based polymers applicable in diverse fields such as drug delivery, gene therapy, combating bacterial and viral infections, creating advanced materials for the food industry, facilitating 3D printing, and even in microelectronics [11].

Chitin, often considered the “forefather” of chitosan, exhibits significantly lower reactivity and poorer chemical versatility due to the absence of a reactive primary amino group [12]. Click reactions in chitin chemistry are scarcely documented, primarily limited to the conventional Cu (I)-catalyzed azide-alkyne dipolar cycloaddition [13]. Recent findings suggest that ultrasound can significantly enhance the reaction rate and yield of target products [14]. Therefore, combining click chemistry with ultrasound presents a promising avenue for advancing chitin chemistry [15].

The phenol-ene click reaction involves the catalytic addition of phenols to double bonds, typically electrophilic olefins [16,17]. This reaction has never been applied in chitin chemistry. In this work, we aim to utilize the phenol-ene reaction to develop new chitin derivatives.

**Scheme 1:**
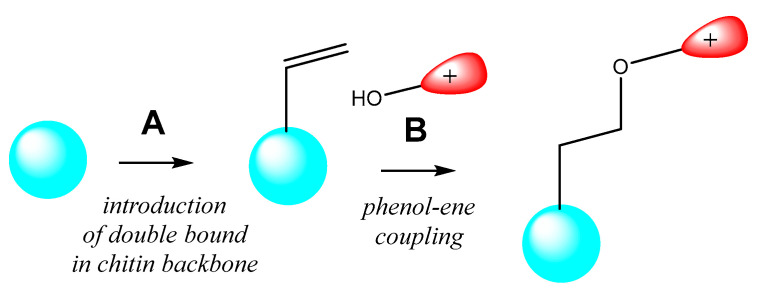
General view of the proposed synthetic route to new cationic chitin derivatives.

Cationic chitin derivatives are known for their significant antibacterial activity, offering a potential alternative to traditional antibiotics [14,18]. In this work, we propose that introducing an alkene function into the chitin molecule (Scheme 1A) and subsequently performing a phenol-ene reaction with a cationic phenolic compound (Scheme 1B) will enable the synthesis of cationic chitin derivatives with antibacterial properties.

It is also very intriguing to determine whether ultrasonication of the reaction mixture can promote and accelerate these reactions. Our work plan also includes assessing the antioxidant activity and toxicity of the resulting polymers. Thus, the aim of this work is to enrich the chemistry of chitin with a new ultrasonic polymer-analogous transformation, synthesize new cationic water-soluble chitin derivatives, and initially evaluate their potential biological properties (antibacterial and antioxidant ones).

## 2. Materials and Methods

### 2.1. Materials

In this study, we used crab shell chitin (Bioprogress, Russia, Moscow) with a viscosity-average molecular weight (MW) 3.4 × 10^4^, 7.2 × 10^4^, 18.8 × 10^4^ Da and degree of acetylation ca. 100%. Acrylic acid, but-3-enoic acid, DMAP (N,N,-dimethylamino pyridine), NHS (*N*-hydroxysuccinimide), EDC (1-ethyl-3-(3-dimethylaminopropyl)carbodiimide), and sodium chloride were purchased from Sigma-Aldrich. N-methyl pyridoxine chloride was kindly provided by Dr. Shakir Abdusalimov (Baku, Azerbaijan). Solvents were obtained from Vecton (Saint Petersburg, Russia). DMAA (dimethylacetamide) was dried and kept under molecular sieve A3. Other solvents were used as received without further purification.

### 2.2. General Methods

Thin-layer chromatography (TLC) was performed on Merck 60 F_254_SiO_2_ plates with a hexane:ethyl acetate from 1:1 to 1:10 (*v*:*v*) mixture as an eluent. The ^1^H NMR spectra were recorded on a Bruker Avance II spectrometer (Karlsruhe, Germany) operating at a frequency of 400 MHz in 1% CF_3_CO_2_H in D_2_O with DOH proton signal suppression. High-resolution electrospray ionization mass spectrometry (positive ion mode) was carried out on a Bruker APEX-Qe ESI FT-ICR instrument (Billerica, MA, USA) with CH_3_CN as a solvent. The apparent hydrodynamic diameter and ζ-potential of nanoparticles in water were estimated at room temperature (about 20 °C) using a Photocor Compact-Z instrument (Moscow, Russia) at λ = 659 nm and θ = 90°. SEM images were obtained by an electron microscope JEOL JSM-6490LV at 15 kV, SEM detector, electron beam size 30, in high vacuum. The test samples were coated with a 20 nm platinum layer in a JEOL auto fine coater JFCS1600 (40 s at 40 mA). Ultrasonic treatments were carried out in an ultrasonic bath ITA (Vitebsk, Belarus), which can work at frequencies of 18, 22, 44 kHz, and 1.5 MHz with a variable power output from 120 to 300 W.

### 2.3. Synthesis of Alkene Chitin Derivatives

Chitin (0.5 g) and lithium chloride (1.4 g) were placed into a vial loaded with a magnetic stirrer bar and the vial was sealed. Next, 30 mL of dry DMAA was injected into the vial and the mixture was purged by dry nitrogen. The mixture was stirred at 20 °C for 3 h to give a pale yellow transparent viscous solution of chitin. Then the vial was opened with a decapper. Then, 0.2, 0.7, or 1 equiv. of but-3-enoic acid, NHS, and EDC, and 0.1 equiv. of DMAP were added to the chitin solution and the reaction mixture was stirred for 3 h at 50 °C. The formed polymers were precipitated by acetone, washed by acetone and ethanol, and dried under vacuum.

### 2.4. Synthesis of Cationic Chitin Derivatives

Alkene chitin derivative (0.5 g) was dissolved in water (50 mL) with vigorous stirring at 70 °C and then the solution was slowly cooled to 20 °C. Then, 0.15, 0,45, or 0.65 equiv. of N-methyl pyridoxine chloride and 0.1 equiv. of DMAP were added to the solution. The resultant mixture was sonicated at 44 kHz, 200 W for 20–25 min. The formed polymers were precipitated by ethanol (100 mL), washed with ethanol (50 mL, three times), and dried in vacuum.

### 2.5. Preparation of Nanoparticles

In total, 20 mg of C-Ch-I-B, C-Ch-II-B, or C-Ch-III-B were dissolved in 20 mL of distilled water and stirred for 3 h. A volume of 25% aqueous sodium tripolyphosphate (TPP) was rapidly added to the polymer solution. The formed nanosuspension was centrifuged at 17,000 rpm and 2 °C for 40 min using an Optima MAX-XP centrifuge (Beckman Coulter, Brea, CA, USA). The dried nanoparticles were redispersed in 15 mL of distilled water and lyophilized.

### 2.6. Biological Experiments

The antibacterial activity in vitro and in vivo and toxicity experiments were performed as described elsewhere [19,20]. 

Animal procedures were compliant with the Ethics Committee and followed the recommendations of European Directive 2010/63/EU of 22 September 20. The ABTS antioxidant test was accomplished according to the standard procedure [21]. The study involved male Wistar rats aged 3 months weighing 180–200 g. All animals were kept in a vivarium under a 24 h photoperiod, controlled temperature (22 ± 2 °C), and air humidity of 65 ± 10% with free access to water and standard feed (granulated compound feed). The experiments were conducted in the first half of the day (10:00–13:00 Moscow time) in compliance with the rules for the humane treatment of laboratory animals. The middle third of the right half of the abdominal wall of white male Wistar rats was shaved, the skin was treated with an alcohol solution of iodine, a microbial mixture was used as an infectious agent, and 3 mL of a polymicrobial suspension were injected into the abdominal cavity in physiological solution. The, 24 h after infection, 200 μL of exudate was collected from the control groups using a sterile syringe. In the experimental groups, a solution of a tested sample was injected to the rats after 24 h. In total, 200 μL of exudate was collected 7 h after the administration. Each obtained exudate was diluted in physiological solution for an hour and 6 ten-fold dilutions of 100 μL were prepared, from which they were applied evenly to a Petri dish with meat-peptone agar. Colonies were counted 24 h after incubation in a thermostat at 37 °C. Subsequently, the number of colony-forming units (CFU) per 1 mL of exudate was recalculated.

The scavenging rate (SR, %) of ABTS cation radicals was calculated according to the equation: SR = [(A_1_ − A_2_)/A_1_] × 100, where A_1_ and A_2_ are the absorbances of ABTS cation radicals at 734 nm before and after addition of the tested sample.

### 2.7. Statistical Analysis

The statistical significance of differences between the samples was determined by one-way analysis of variance (ANOVA) using JMP 5.0.1 software (SAS Campus Drive, Cary, NC, USA). Mean values, where appropriate, were compared by Student’s *t*-test at a significance level of *p* < 0.05.

## 3. Results and Discussion

### 3.1. Synthesis of Cationic Chitin Derivatives by Ultrasonic Phenol-Ene Reaction

**Scheme 2:**
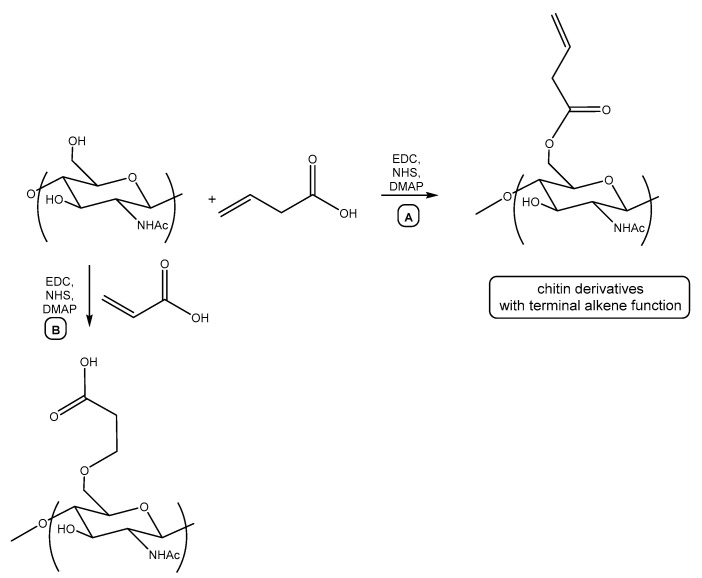
Synthesis of alkene derivatives of chitin.

To introduce a double bond into chitin macromolecule, we used treatment of chitin with acrylic acid in presence of EDC, NHS, and DMAP hoping to achieve ester bond formation by the classical carbodiimide method, which provides chemical interaction between the carboxyl group of acrylic acid and the hydroxyl group of chitin at position *6* (Scheme 2B) [22]. We found that in the temperature range of 20–40 °C this interaction is not feasible. As the temperature increases, the Michael reaction occurs furnishing carboxyethyl chitin derivatives. Apparently, this is due to the high electrophilicity of the double bond in the acrylic acid molecule, which ensures that the process occurs along the Michael reaction route [23], and not through the interaction of the carboxyl group with the hydroxyl functionality.

**Figure 1:**
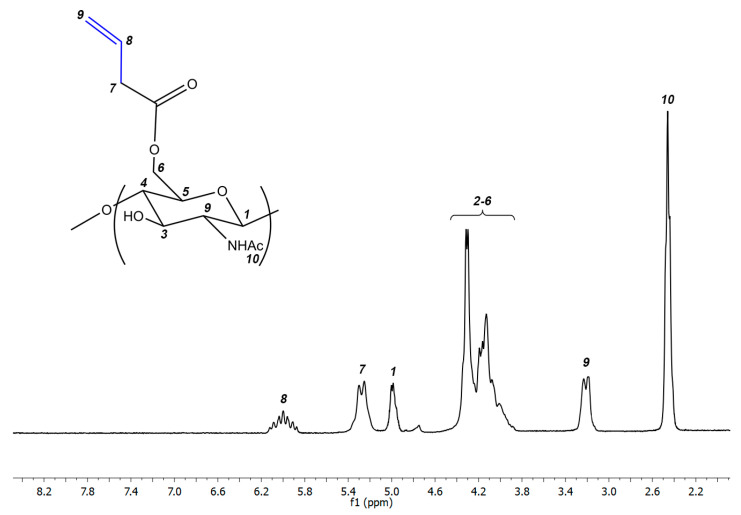
^1^H NMR spectrum A-Ch-III-B.

In this regard, we decided to use but-3-enoic acid as a reactant, which contains a much less electrophilically activated C=C double bond. The interaction of chitin with but-3-enoic acid in the presence of EDC, NHS, and DMAP leads to the formation of the desired alkene chitin derivatives bearing a double bond (Scheme 2A). This reaction proceeds almost identically for chitin of high, medium, and low molecular weight, and the degree of substitution of the resulting chitin derivatives is easily regulated by changing the but-3-enoic acid/chitin molar ratio. Thus, we synthesized chitin derivatives of low, medium, and high molecular weight with low, moderate, and high degrees of substitution bearing a terminal C=C double bond (Table 1). The resulting polymers were characterized using ^1^H NMR spectroscopy. A typical spectrum with signal assignment is shown in Figure 1. The degree of substitution of the alkene chitin derivatives was calculated according to the formula: DS = I (H8) = I (H7)/2 = I (H9)/2, when I (H1) = 1. In the formula above I (HX)–integral intensity of the signal of proton X.

**Scheme 3:**
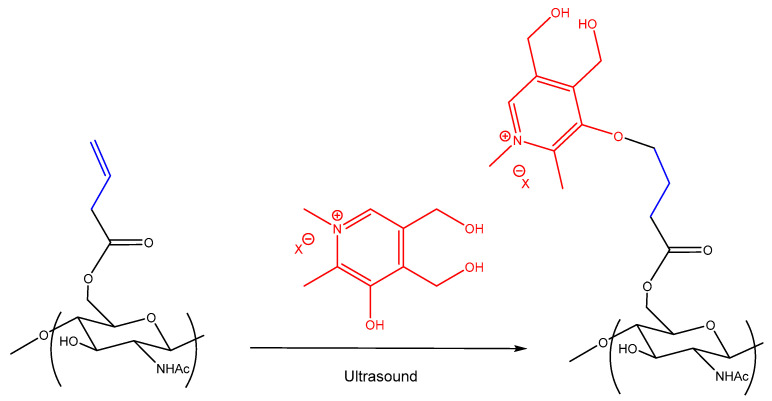
Synthesis of cationic chitin derivatives by phenol-in reaction.

At the next stage, the alkene derivatives of chitin were involved in phenol-ene addition reaction with an N-methylated derivative of the natural vitamin pyridoxine (Scheme 3). This reaction does not occur even at 70 °C but becomes feasible under DMAP catalyst, which converts the phenolic group into a much more reactive nucleophilic phenolate ion. However, even in this case, we were not able to achieve complete conversion of the starting alkene derivatives of chitin into the desired products.

We used the found optimal acoustic conditions (44 kHz, 200 W) to promote the macromolecular phenol-ene reaction (Scheme 3).

**Scheme 4:**
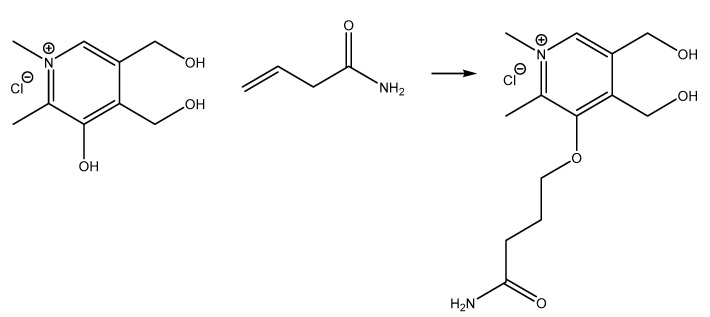
Model phenol-yne click reaction.

We were inspired by our previous findings on the high efficiency of ultrasound for intensifying azide-alkyne cycloaddition in chitin chemistry [13,24]. We intended to use ultrasound treatment of the model DMAP-catalyzed phenol-ene reaction presented in Scheme 4. At the preliminary stage of our work, we were forced to optimize the acoustic conditions (ultrasound frequency and power) for the phenol-ene reaction between low molecular weight partners. We used N-methylpyriodoxine (phenolic component) and but-3-enoic acid amide (enoic component) as reagents to find the best ultrasonic parameters (Scheme 4). Experiments were carried out in cavitation mode at frequencies of 18, 22, 44 kHz, and 1.5 MHz, and powers of 120–300 W, using thin layer chromatography and high-resolution mass spectrometry (electrospray ionization) to monitor the reaction. The frequency and power of ultrasonic irradiation have a pronounced effect on the rate and selectivity of the reaction. We found that the model reaction was optimally intensified at a frequency of 44 kHz and a power of 200 W, offering 100% conversion of starting materials to the phenol-ene coupling product in 20 min. The application of lower frequencies and powers naturally reduces the rate of the model reaction, while increasing the frequency to 1.5 MHz at any power causes the reaction to lose its selectivity and results in a wide mixture of unidentified compounds among the main product (seven spots on TLC).

We used the found optimal acoustic conditions (44 kHz, 200 W) to promote the macromolecular phenol-ene reaction (Scheme 3).

**Figure 2:**
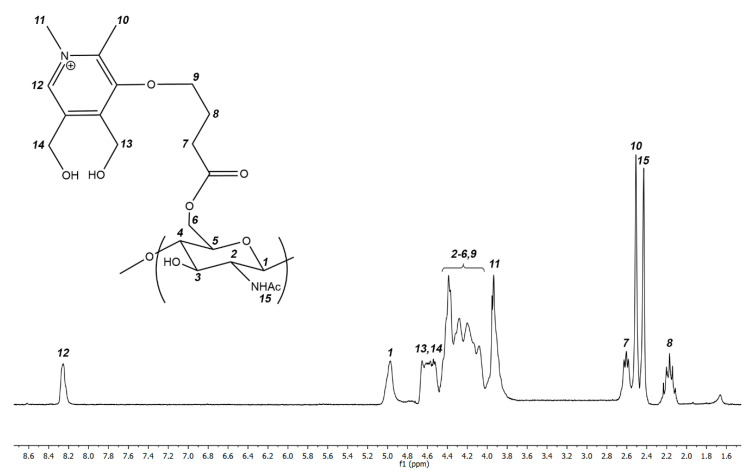
^1^H NMR spectrum C-Ch-III-B.

The reaction proceeds selectively to give rise the desired cationic chitin derivatives within 20–25 min. The degree of substitution of cationic polymers coincides with the degree of substitution of the original alkene chitin derivatives. The products are easily isolated from the reaction mixture by simple precipitation followed by washing, and their purification does not require a lengthy dialysis procedure. The resulting polymers are readily soluble in water over the entire pH range. Thus, we have obtained water-soluble cationic chitin derivatives of high, medium, and low molecular weight with high, moderate, and medium degrees of substitution. A typical spectrum of a cationic polymer is presented in Figure 2. The disappearance of proton resonances at the C=C double bond (signals of protons 9 and 8 in Figure 1), as well as the equality of the degrees of substitution of the starting alkene derivative and the final product, indicates complete conversion of the alkene chitin species into a cationic polymer. Degree of substitution of the cationic chitin derivatives was calculated according to the formula: DS = I (H12), when I (H1) = 1. In the formula above I (HX)–integral intensity of the signal of proton X.

Water-soluble chitosan derivatives are widely described in the literature; there are more than a hundred of them [25,26], while the literature reports significantly fewer water-soluble chitin derivatives (more specifically, less 30% substituted tosylated chitin [27], aminated chitin [28], PTMA-substituted chitin [29,30] hydroxypropyl chitin [31], chitin phosphate [32], diethylaminoethyl chitin [33], carboxymethylated [34] and succinylated chitin derivatives [35], and a few others). The synthesis of a new water-soluble chitin derivative is a highlight in the polysaccharide field of polymer science, and we were fortunate to add a new type of chitin-based polymer to this treasure trove.

### 3.2. Preparation of Nanoparticles of Cationic Chitin-Based Polymer

In many instances, converting polymers from their native state (e.g., macromolecular coil conformation) to nanoparticle form proves to be a very effective tool for improving their physicochemical or biological properties [36]. For example, chitosan nanoparticles, unlike the original chitosan, turn out to be more effective catalysts for a number of organic transformations [37,38]. Triazolbetaine chitin derivatives in form of heir nanoparticles exhibit significantly more pronounced antibacterial and antifungal effect than starting polymers [13]. These facts encouraged us to attempt to obtain nanoparticles of cationic chitin derivatives.

Ionic gelation is perhaps the most common method for producing nanoparticles based on natural polysaccharides and their derivatives [39]. This method does not require special equipment and harsh conditions (compared, for example, with spray-drying), which ensures the integrity of the polymer chains. In addition, the size and the zeta potential of the resulting nanoparticles can often be easily controlled by changing the amount of gelling reagent [40]. In this work, we chose the ionic gelation technique to obtain nanoparticles of cationic chitin derivatives, and used sodium tripolyphosphate (TPP) as the gelling agent.

The electrostatic interaction of a cationic derivative with the TPP anion leads to the formation of nanoparticles (aggregates) with a size of ca. 100–400 nm. In the case of polymers derived from low molecular weight chitin, we were unable to optimize the conditions for the synthesis of nanoparticles with a unimodal size distribution. In the case of high molecular weight chitin derivatives, large aggregates are formed (that is, a precipitate occurs). For chitin derivatives of medium molecular weight, we were able to optimize the ionic gelation conditions and prepare nanoparticles with a unimodal size distribution. The hydrodynamic diameter of the resulting nanoparticles depends on the polymer/TPP ratio (Table 2).

An increase in the amount of added TPP first leads to a decrease in the hydrodynamic diameter of the resulting particles to ca. 100 nm, since the attractive forces between opposite charges (cationic polymer and an increasing amount of anionic TPP) lead to compaction of the resulting particles. Further addition of TPP leads to an increase in the size of the nanoparticles. Apparently, this is due to the emergence of repulsive forces between negative charges due to an excess of TPP. The influence of the amount of added TPP on the zeta potential is also natural. Increasing the amount of anionic TPP reduces the zeta potential of the resulting particles.

**Figure 3:**
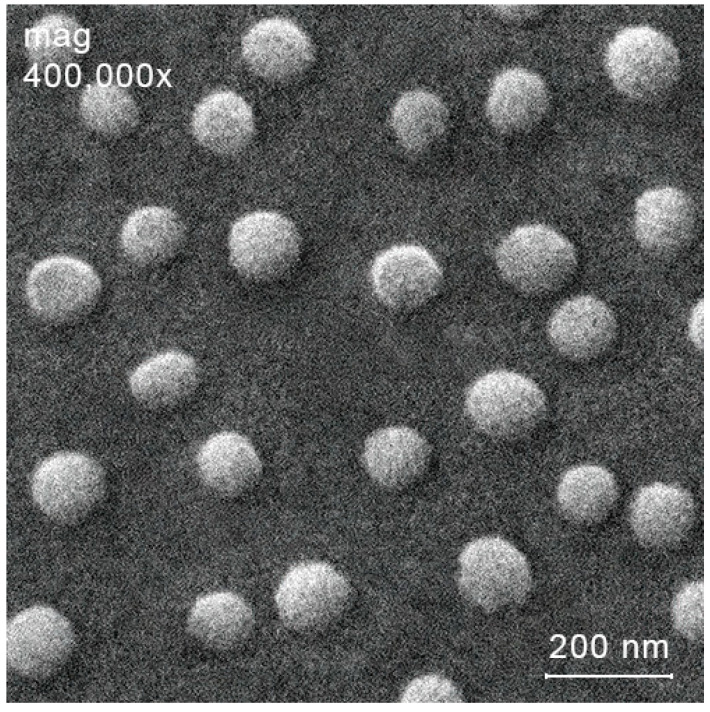
SEM image of NP-3-III.

The resulting nanoparticles are characterized by a unimodal size distribution and spherical morphology, which was also confirmed using scanning electron microscopy (SEM). As an example, an SEM image of the most biologically active nanoparticles (see Section 3.4) NP-3-III is presented in Figure 3. 

### 3.3. Antibacterial Activity and Toxicity

We intended to prepare antibacterial derivatives from almost 100% acetylated chitin, practically inactive in antibacterial terms, by introducing a cationic substituent into the polymer chain. After synthesizing and characterizing cationic chitin derivatives, we assessed their in vitro antibacterial activity using the conventional agar diffusion method [41]. A quantitative indicator in the agar assay is the diameter of the bacterial growth inhibition zone. The most active antibacterial compounds cause the largest inhibition zone. The obtained experimental data testing the antibacterial effect of cationic chitin derivatives and their based nanoparticles are summarized in Table 3.

The antibacterial activity of polymers depends dramatically on their degree of substitution and increases with an increasing degree of substitution. For example, low-substituted (degree of substitution about 0.15) derivatives of high molecular weight chitin C-Ch-I-C cause the growth inhibition zone of *Bacillus subtilis* of about 13.5 mm, while its highly substituted analogue (degree of substitution about 0.65) C-Ch-III-C provokes an inhibition circle diameter 19.1 mm. The same pattern is observed for *Klebsiella pneumoniae*. The increase in the antibacterial effect with increasing degree of substitution is explained by an increase in the cationic density of the polymer, since the substituent introduced into the side chain bears a positively charged quaternized nitrogen atom [42]. 

In all cases, antibacterial activity of the tested samples against *Bacillus subtilis* was superior to that against *Klebsiella pneumoniae*. This fact is not surprising, since *Klebsiella pneumoniae* is a Gram-negative microorganism that is much more resistant to antibacterial agents, since it contains a damage-resistant cell wall that two (internal and external) membranes functioning as double protection from the antibacterial agent [43].

The molecular weight of polymers also has a very noticeable, although not pronounced, effect on their antibacterial activity. Without exception, polymers with medium molecular weight are, albeit slightly, more effective than their low or high molecular-weight counterparts. For example, among highly substituted polymers, the medium molecular weight chitin derivative C-Ch-III-B provokes a larger zone of inhibition for *Bacillus subtilis* (22.3 mm) than the low and high molecular weight analogues C-Ch-III-A and C-Ch-III-C (*Bacillus subtilis* inhibition zone 19.3 and 19.1, respectively). To explain this not entirely clear fact, additional biological studies are required, possibly involving fluorescent labels and confocal microscopy, as well as elucidation of the detailed mechanisms of antibacterial action. However, this is not the first time we have encountered such examples of the manifestation of an antibacterial effect in medium molecular weight polymers, and we described them earlier [20,44].

Since derivatives of medium molecular weight chitin exhibit the greatest antibacterial effect, we used these polymers to prepare their nanoparticles (Section 3.2). The antibacterial activity of the synthesized nanoparticles depends mainly on three factors: (i) degree of substitution of the starting polymer, (ii) size of nanoparticles, and (iii) their zeta potential (see Table 2 and Table 3). It is quite expected that the most active antibacterial nanoparticles are those prepared from the most antibacterial active polymer, i.e., highly substituted C-Ch-III-B. Nanoparticles derived from less active moderate- and low-substituted analogues C-Ch-II-B and C-Ch-I-B demonstrate a significantly lower antibacterial effect. In all cases, the most effective antibacterial agents are nanoparticles characterized by combination of minimum size and maximum positive zeta potential. For example, among nanoparticles derived from C-Ch-III-B, NP-3-III are the most active (they are characterized by a minimum size with a fairly high zeta potential). The data in Table 2 and Table 3 lead us to assume that among these two factors, the most important is the minimum nanoparticle size. Probably, nanoparticles have higher antibacterial activity than the corresponding chitin derivatives since the spatial orientation (or conformation) of the polymers in nanoparticles is more favorable for interaction with the bacterial cell wall, which means that cations are better exposed on the nanoparticle surface for attractive electrostatic interactions with negatively charged bacterial cell walls. Also, a certain contribution to the development of the antibacterial effect can be due to hydrophobic interactions between chitin derivatives and bacterial cell walls [20].

In general, in vitro antibacterial activity studies allowed us to conclude that among the tested polymers the most active is C-Ch-III-B, and among nanoparticles NP-3-III. These chitin-based systems were used for further in vivo study of their antibacterial effect and toxicity (10 rats for treated group and 10 rats for control group). The in vivo antibacterial activity of chitin-based systems was assessed in rats subjected to model peritonitis by infection with a microbial mixture [45]. After 24 h of single infection, the animals showed full-blown symptoms of acute peritonitis, and we began treating them with C-Ch-III-B polymer, NP-3-III nanoparticles, or the commercial antibiotic ampicillin. For the control group, we injected the rats with isotonic NaCl solution instead of treatment. At 7 h after injection (i.e., 31 h after infection), we collected exudate from the peritoneal cavity of animals and quantified the bacterial content in the exudate by the number of colony-forming units per milliliter of exudate (CFU/mL). Ampicillin quite effectively reduces the CFU/mL value (up to 108 CFU/mL) compared to the control (3015 CFU/mL). In the case of using C-Ch-III-B polymer and NP-3-III nanoparticles to treat infected rats, we did not detect CFU at all in the peritoneal exudate. Thus, the in vivo experiments on the rat model of peritonitis demonstrated that neither C-Ch-III-B nor NP-3-III is inferior in its effectiveness to the commercial antibiotic ampicillin.

We assessed the in vivo toxicity of the leading chitin-based antibacterial systems, i.e., polymer C-Ch-III-B and its nanoparticles NP-3-III also on uninfected healthy rats by injection according to the standard unified procedure, which we have used for related polysaccharide species in [46]. For comparison, we used the chitin-derived polymer chitosan, which is considered a non-toxic, water-soluble derivative of chitin in its hydrochloride form.

We found that administration of C-Ch-III-B, NP-3-III, or chitosan had the same effect and did not result in any deterioration in the condition of healthy rats. The animals did not show any signs of acute intoxication. We found that no experimental animals died from the administered dose. This fact allowed us to conclude that the LD_50_ exceeds 2000 mg/kg, which in turn means that C-Ch-III-B and NP-3-III, as well as chitosan, can be confidently classified as category IV (low toxic compounds). During the experiment, we detected the complete normal behavior of rats (normal eating and drinking, normal coordination of movements, no disturbances in the sleep/wake cycle, frequency and depth of breathing, heart rate not exceeding the norm, no deviations in defecation and urination from the norm).

Thus, we have found new highly active antibacterial chitin derivatives, which are not inferior to ampicillin in their in vivo effectiveness, and at the same time are non-toxic. These results can be considered as some of the best in the field of antibacterial chitin derivatives so far reported in the literature [47].

### 3.4. Antioxidant Activity 

Oxidative stress causes or at least accompanies many pathological processes in cells, tissues, and organs of mammals [48,49,50]. Oxidative stress is a nonspecific pathological process and leads to the accumulation of reactive oxygen species in cells and interstitial fluids [48]. Reactive oxygen species can damage intracellular organelles, DNA, RNA, and other essential components of cells. This leads to inevitable accumulation of mutations, an increase in likelihood of carcinogenesis, uncoupling of respiration and phosphorylation, and other cascades of unfavorable processes [48]. Antioxidants act as protection against reactive oxygen species, since they trap reactive oxygen [51]. The search for new highly effective antioxidants, as well as compounds that, in addition to the main pharmacological effect, also exhibit antioxidant activity, is an important area in medicinal chemistry [52].

**Figure 4:**
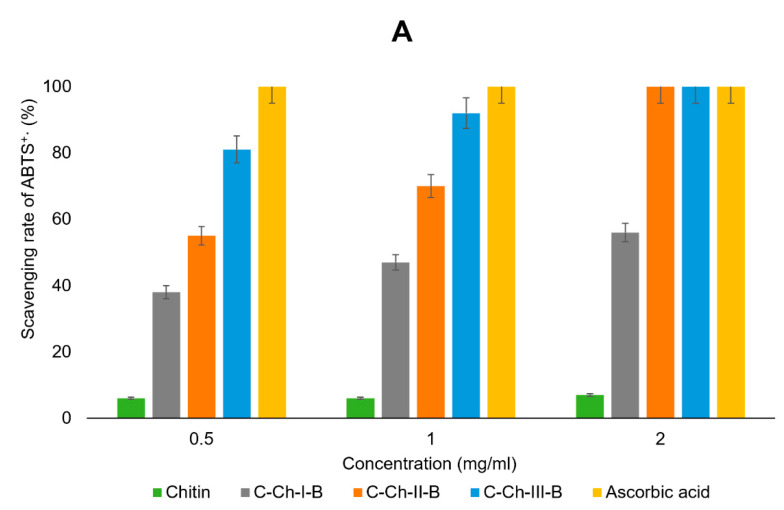

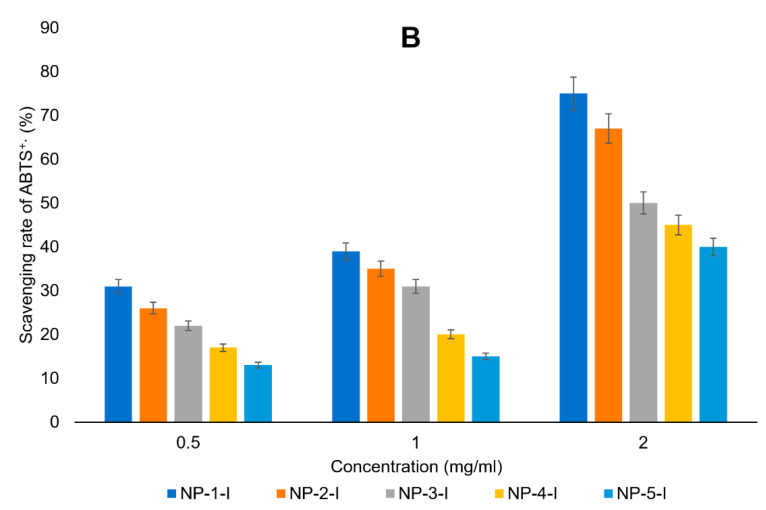
Antioxidant activity of polymers (**A**) and their nanoparticles (**B**).

Pyridoxine and many other compounds, such as gallic and ellagic acid, which contain an oxygen atom directly bonded to the phenyl ring, often exhibit high antioxidant activity [53,54]. The cationic derivatives we obtained bear a pyridoxine-derived substituent, and this allowed us to assume that the synthesized polymers have an antioxidant effect (since the pyridoxine moiety is an electron-rich substituent, which can act as a reducing agent to oxidation–reduction reactions’ electron donors) [54]. We assessed the antioxidant activity of the resulting systems (polymers and their nanoparticles) using the classical method based on their ability to capture ABTS^+^• radical cations [55]. We compared the antioxidant activity of the synthesized systems with that of one of the most powerful natural antioxidants, i.e., ascorbic acid, as well as starting chitin. For the conventional test, we measured the antioxidant activity of samples at concentrations of 0.5, 1, and 2 mg/mL. In the preliminary stages of our experiment, we found that the antioxidant effect does not depend on the molecular weight of the tested polymer. Thus, in the current paragraph we discuss the antioxidant effect of polymer systems with medium molecular weight, since they have proven to be the most active antibacterial agents (see Section 3.3). The results of this study are presented in Figure 4.

At a concentration of 0.5 mg/mL, only ascorbic acid is capable of scavenging 100% of ABTS^+^• radical cations and it demonstrates the most powerful antioxidant effect. Starting chitin at the same concentration exhibits the least antioxidant effect (ABTS^+^• radical cation capture about 6%). The extremely low effect of chitin can be explained by its complete insolubility in water. Chitin derivatives have a greater antioxidant effect than the starting polymer (Figure 4A). Their antioxidant activity increases with increasing degree of substitution (ABTS^+^• radical cation capture of about 38% for C-Ch-I-B, about 55% for C-Ch-II-B, and about 81% for C-Ch-III-B). The same patterns of the antioxidant activity of polymers depending on the degree of substitution are also observed for other sample concentrations. An increase in the concentration of samples naturally results in an increase in their antioxidant activity. For example, at a concentration of 1 mg/mL, C-Ch-III-B is able to capture 92% of ABTS^+^• radical cations; when the concentration of C-Ch-III-B increases to 2 mg/mL, the capture efficiency increases to 100%.

Conversion of polymers into corresponding nanoparticles leads to a decrease in antioxidant activity (Figure 4B). The antioxidant activity of nanoparticles directly depends on the amount of TPP added to the corresponding polymer to prepare nanoparticles. For example, antioxidant activity decreases markedly in the series NP-1-I–NP-2-I–NP-3-I–NP-4-I–NP-5-I, obtained from C-Ch-I-B by adding increasing amount of TPP (see Table 2). This, apparently, can be explained by the increasing degree of compaction of nanoparticles and the strong electrostatic interaction of the anionic sterically large TPP with the cationic substituent, which provides an antioxidant effect. Both factors contribute to a decrease in the availability of the side substituent and, therefore, a decrease in its reactivity in redox transformations.

In addition, we compared the IC_50_ values for the test samples with the IC_50_ of ascorbic acid. IC_50_ is an indicator of the concentration of antioxidant required for 50% ABTS+• radical cation in vitro. The data obtained (Table 4) are consistent with those obtained when comparing the antioxidant effect of samples with different concentrations.

Among obtained chitin-based systems, the lowest IC_50_ value is characteristic of C-Ch-III-B, and this corresponds to its maximum antioxidant effect among the tested polymers and their nanoparticles.

## 4. Conclusions

The results of this work can be summarized in the following key points:

First, we conducted the first ultrasonic phenol-ene addition and utilized the phenol-ene coupling in polymer chemistry as a polymer-analogous transformation. This approach enabled the synthesis of water-soluble chitin derivatives with preparative simplicity and convenience. Compared to water-soluble chitosan derivatives, water-soluble derivatives of fully deacetylated chitin are scarcely documented. Therefore, the synthesis of each new type of water-soluble chitin derivative is a significant advancement in the field of chitinology.

Secondly, we assessed the antibacterial activity of the synthesized cationic chitin derivatives and found that it depends on both the molecular weight and the degree of substitution. Polymers of medium molecular weight exhibited the greatest antibacterial effect in each series, and this effect increased with a higher degree of substitution. Transforming these polymers into their nanoparticle form can result in either an increase or decrease in antibacterial activity. The greatest antibacterial effect was observed for nanoparticles with the smallest size and a sufficiently high positive zeta potential.

These findings allowed us to identify leading antibacterial systems, specifically polymer C-Ch-III-B and its derived nanoparticles NP-3-III. In in vivo experiments on rats, these systems demonstrated an antibacterial effect comparable to that of the commercial antibiotic ampicillin, while also showing no signs of toxicity. These results are among the most promising in the field of antibacterial chitin derivatives reported to date. Undoubtedly, these antibacterial systems are highly interesting and warrant further preclinical studies.

Thirdly, the resulting polymers (unlike their nanoparticles) exhibited a pronounced antioxidant effect, which is independent of the polymer’s molecular weight. This effect is attributed to the substituent introduced into the side chain and increases with the degree of substitution. The most potent antioxidant effect was observed in the most effective antibacterial polymer, C-Ch-III-B.

Finally, we have once again demonstrated the power and elegance of ultrasound in polymer chemistry, underscoring the significance of the sonochemical approach in the chemical modification of polysaccharides. This technique facilitates the synthesis of derivatives with enhanced physicochemical and biological properties.

The authors are clearly aware of some limitations of this study. Since the main part of this article and its novelty are focused on the chemistry and new polymer-analogous transformation of chitin, the main limitations concern the biological properties of the synthesized polymers. In this work, we present and discuss only a primary assessment of the antioxidant and antibacterial activity of chitin derivatives. Of course, for a more in-depth analysis of biological effects, understanding the mechanisms of their implementation, further thorough biological studies are necessary, and this project is underway in our group.

## Figures and Tables

**Table 1:** Alkene and cationic chitin derivatives.

Name of Alkene Chitin Derivative	Name of Cationic Chitin Derivative	Molecular Weight of the Original Chitin	Degree of Substitution of Chitin Derivative
A-Ch-I-A	C-Ch-I-A	3.4 × 104	0.15
A-Ch-I-B	C-Ch-I-B	7.2 × 104	0.15
A-Ch-I-C	C-Ch-I-C	18.8 × 104	0.15
A-Ch-II-A	C-Ch-II-A	3.4 × 104	0.46
A-Ch-II-B	C-Ch-II-B	7.2 × 104	0.44
A-Ch-II-C	C-Ch-II-C	18.8 × 104	0.45
A-Ch-III-A	C-Ch-III-A	3.4 × 104	0.67
A-Ch-III-B	C-Ch-III-B	7.2 × 104	0.66
A-Ch-III-C	C-Ch-III-C	18.8 × 104	0.65

**Table 2:** Hydrodynamic diameter and zeta potential of nanoparticles.

Type of Nanoparticles	Mean Hydrodynamic Diameter, nm *	TPP:Polymer Mass Ratio	V(TPP), mL *	Polydispersity Index *	ζ-Potential, mV *
Prepared from C-Ch-I-B					
NP-1-I	400 ± 5	1:228	0.35	0.11 ± 0.02	53.1 ± 0.1
NP-2-I	202 ± 7	1:94	0.85	0.11 ± 0.05	48.3 ± 0.2
NP-3-I	108 ± 4	1:67	1.20	0.10 ± 0.03	42.7 ± 0.3
NP-4-I	312 ± 4	1:55	1.45	0.13 ± 0.04	33.4 ± 0.1
NP-5-I	450 ± 6	1:47	1.70	0.15 ± 0.02	25.2 ± 0.2
Prepared from C-Ch-II-B					
NP-1-II	407 ± 4	1:133	0.60	0.13 ± 0.03	52.3 ± 0.3
NP-2-II	211 ± 8	1:76	1.05	0.14 ± 0.03	46.8 ± 0.1
NP-3-II	96 ± 3	1:50	1.60	0.11 ± 0.02	41.2 ± 0.4
NP-4-II	303 ± 9	1:43	1.85	0.12 ± 0.03	30.9 ± 0.2
NP-5-II	472 ± 4	1:39	2.05	0.11 ± 0.03	24.0 ± 0.1
Prepared from C-Ch-III-B					
NP-1-III	390 ± 9	1:80	1.00	0.10 ± 0.04	50.7 ± 0.2
NP-2-III	201 ± 4	1:57	1.40	0.12 ± 0.02	46.1 ± 0.5
NP-3-III	110 ± 6	1:50	1.60	0.10 ± 0.04	44.4 ± 0.3
NP-4-III	312 ± 3	1:35	2.30	0.11 ± 0.01	32.5 ± 0.1
NP-5-III	457 ± 3	1:33	2.45	0.11 ± 0.02	23.0 ± 0.3

* Mean value ± SD, *n* = 3

**Table 3:** Antibacterial activity of the tested systems.

Sample	*Bacillus subtilis*	*Klebsiella pneumoniae*
	Inhibition Zone, mm *	
Ampicillin	30.6 ± 0.2	23.3 ± 0.2
Chitin	Effect Not Detected	Effect Not Detected
Polymers		
C-Ch-I-A	14.0 ± 0.3	11.5 ± 0.3
C-Ch-I-B	15.7 ± 0.4	12.7 ± 0.1
C-Ch-I-C	13.5 ± 0.1	11.2 ± 0.4
C-Ch-II-A	16.2 ± 0.3	15.1 ± 0.1
C-Ch-II-B	18.1 ± 0.1	16.6 ± 0.3
C-Ch-II-C	15.3 ± 0.1	14.2 ± 0.3
C-Ch-III-A	19.3 ± 0.2	18.2 ± 0.2
C-Ch-III-B	22.3 ± 0.3	19.7 ± 0.2
C-Ch-III-C	19.1 ± 0.2	17.5 ± 0.1
Nanoparticles derived from C-Ch-I-B		
NP-1-I	15.9 ± 0.2	13.4 ± 0.2
NP-2-I	17.1 ± 0.4	14.1 ± 0.2
NP-3-I	19.3 ± 0.3	16.2 ± 0.4
NP-4-I	14.0 ± 0.1	13.2 ± 0.2
NP-5-I	12.5 ± 0.3	12.5 ± 0.3
Nanoparticles derived from C-Ch-II-B		
NP-1-II	18.8 ± 0.1	17.8 ± 0.1
NP-2-II	20.1 ± 0.2	19.3 ± 0.3
NP-3-II	23.6 ± 0.1	21.7 ± 0.2
NP-4-II	21.3 ± 0.1	19.1 ± 0.2
NP-5-II	19.5 ± 0.3	18.4 ± 0.3
Nanoparticles derived from C-Ch-III-B		
NP-1-III	24.4 ± 0.1	20.5 ± 0.3
NP-2-III	26.9 ± 0.1	22.2 ± 0.2
NP-3-III	30.8 ± 0.4	24.8 ± 0.2
NP-4-III	26.5 ± 0.3	19.6 ± 0.1
NP-5-III	23.2 ± 0.2	17.3 ± 0.3

* Mean value ± SD (*n* = 3).

**Table 4:** IC50 values of the tested samples.

Sample	IC50, mg/mL *	Sample	IC50, mg/mL *
C-Ch-I-B	1.12 ± 0.02	NP-3-I	2.05 ± 0.03
C-Ch-II-B	0.90 ± 0.03	NP-4-I	1.48 ± 0.02
C-Ch-III-B	0.29 ± 0.01	NP-5-I	0.63 ± 0.03
NP-1-I	2.41 ± 0.04	Ascorbic acid	0.016 ± 0.003
NP-2-I	2.25 ± 0.03		

* Mean value ± SD (*n* = 3).

## Data Availability

Data are contained within the article.

## References

[1] Stump B. (2022). Click Bioconjugation: Modifying Proteins Using Click-Like Chemistry. ChemBioChem.

[2] Halay E., Acikbas Y. (2023). Click chemistry: A fascinating, Nobel-winning method for the improvement of biological activity. Appl. Chem. Eng..

[3] Fantoni N.Z., El-Sagheer A.H., Brown T. (2021). A Hitchhiker’s Guide to Click-Chemistry with Nucleic Acids. Chem. Rev..

[4] Devaraj N.K., Finn M.G. (2021). Introduction: Click Chemistry. Chem. Rev..

[5] Spruell J.M., Spruell J.M. (2011). Efficient Templated Synthesis of Donor–Acceptor Rotaxanes Using Click Chemistry. The Power of Click Chemistry for Molecular Machines and Surface Patterning.

[6] Chandrasekaran S. (2016). Click Reactions in Organic Synthesis.

[7] Kaur J., Saxena M., Rishi N. (2021). An Overview of Recent Advances in Biomedical Applications of Click Chemistry. Bioconjug. Chem..

[8] Kou S.G., Peters L., Mucalo M. (2022). Chitosan: A review of molecular structure, bioactivities and interactions with the human body and micro-organisms. Carbohydr. Polym..

[9] Chatterjee R., Maity M., Hasnain M.S., Nayak A.K. (2021). Chitosan: Source, chemistry, and properties. Chitosan in Drug Delivery.

[10] Cheaburu-Yilmaz C.N., Karavana S.Y., Yilmaz O., Lepadatescu B. (2017). Functionalization of Chitosan by Click Chemistry.

[11] Kritchenkov A.S., Skorik Y.A. (2017). Click reactions in chitosan chemistry. Russ. Chem. Bull..

[12] Lv J., Lv X., Ma M., Oh D.H., Jiang Z., Fu X. (2023). Chitin and chitin-based biomaterials: A review of advances in processing and food applications. Carbohydr. Polym..

[13] Liu Y., Li B., Xu C., Shi Z., Liu L., Fan Y., Yu J. (2024). Efficient preparation of fluorescent nanomaterials derived from chitin via a modification-first strategy assisted by click chemistry. Green Chem..

[14] Kritchenkov A.S., Egorov A.R., Abramovich R.A., Kurliuk A.V., Shakola T.V., Kultyshkina E.K., Ballesteros Meza M.J., Pavlova A.V., Suchkova E.P., Le Nhat Thuy G. (2021). Water-soluble triazole chitin derivative and its based nanoparticles: Synthesis, characterization, catalytic and antibacterial properties. Carbohydr. Polym..

[15] Kritchenkov A.S., Kletskov A.V., Egorov A.R., Kurasova M.N., Tskhovrebov A.G., Khrustalev V.N. (2021). Ultrasound and click chemistry lead to a new chitin chelator. Its Pd(II) complex is a recyclable catalyst for the Sonogashira reaction in water. Carbohydr. Polym..

[16] Hu Y., Dziekonski E.T., Wang D.M., Gonzalez L.E., Cooks R.G. (2024). Rapid Identification of Phenolics in Mixtures by Two-Dimensional Tandem Mass Spectrometry with Microdroplet Accelerated Derivatization Reactions. Anal. Sens..

[17] Zhang J., Niu D., Brinker V.A., Hoye T.R. (2016). The Phenol-Ene Reaction: Biaryl Synthesis via Trapping Reactions between HDDA-Generated Benzynes and Phenolics. Org. Lett..

[18] Varlamov V.P., Il’ina A.V., Shagdarova B.T., Lunkov A.P., Mysyakina I.S. (2020). Chitin/Chitosan and Its Derivatives: Fundamental Problems and Practical Approaches. Biochemistry.

[19] Sahm D.H., Murray P.R. (1991). Antibacterial susceptibility tests: Dilution methods. Manual of Clinical Microbiology.

[20] Kritchenkov A.S., Zhaliazniak N.V., Egorov A.R., Lobanov N.N., Volkova O.V., Zabodalova L.A., Suchkova E.P., Kurliuk A.V., Shakola T.V., Rubanik V.V. (2020). Chitosan derivatives and their based nanoparticles: Ultrasonic approach to the synthesis, antimicrobial and transfection properties. Carbohydr. Polym..

[21] Re R., Pellegrini N., Proteggente A., Pannala A., Yang M., Rice-Evans C. (1999). Antioxidant activity applying an improved ABTS radical cation decolorization assay. Free Radic. Biol. Med..

[22] Gürer F., Kargl R., Bračič M., Makuc D., Thonhofer M., Plavec J., Mohan T., Kleinschek K.S. (2021). Water-based carbodiimide mediated synthesis of polysaccharide-amino acid conjugates: Deprotection, charge and structural analysis. Carbohydr. Polym..

[23] Sashiwa H., Yamamori N., Ichinose Y., Sunamoto J., Aiba S.I. (2003). Michael reaction of chitosan with various acryl reagents in water. Biomacromolecules.

[24] Kritchenkov A.S., Egorov A.R., Volkova O.V., Artemjev A.A., Kurliuk A.V., Anh Le T., Hieu Truong H., Le-Nhat-Thuy G., Van Tran Thi T., Van Tuyen N. (2021). Novel biopolymer-based nanocomposite food coatings that exhibit active and smart properties due to a single type of nanoparticles. Food Chem..

[25] Wan Yusof W.R., Awang N.Y.F., Azhar Laile M.A., Azizi J., Awang Husaini A.A.S., Seeni A., Wilson L.D., Sabar S. (2023). Chemically modified water-soluble chitosan derivatives: Modification strategies, biological activities, and applications. Polym.-Plast. Technol. Mater..

[26] Kahya N. (2019). Water Soluble Chitosan Derivatives and their Biological Activities: A Review. Polym. Sci..

[27] Kurita K., Inoue S., Nishimura S.-I. (1991). Preparation of soluble chitin derivatives as reactive precursors for controlled modifications: Tosyl- and iodo-chitins. J. Polym. Sci. Part A Polym. Chem..

[28] Pourjavadi A., Seidi F., Salimi H. (2007). Synthesis of Novel Water-Soluble Aminodeoxychitin Derivatives. Starch-Stärke.

[29] Peng N., Ai Z., Fang Z., Wang Y., Xia Z., Zhong Z., Fan X., Ye Q. (2016). Homogeneous synthesis of quaternized chitin in NaOH/urea aqueous solution as a potential gene vector. Carbohydr. Polym..

[30] Ding F.Y., Shi X.W., Li X.X., Cai J., Duan B., Du Y.M. (2012). Homogeneous synthesis and characterization of quaternized chitin in NaOH/urea aqueous solution. Carbohydr. Polym..

[31] Park I.K., Park Y.H. (2001). Preparation and structural characterization of water-soluble O-hydroxypropyl chitin derivatives. J. Appl. Polym. Sci..

[32] Nishi N., Nishimura S.-I., Ebina A., Tsutsumi A., Tokura S. (1984). Preparation and characterization of water-soluble chitin phosphate. Int. J. Biol. Macromol..

[33] Kurita K., Koyama Y., Inoue S., Nishimura S. (1990). ((Diethylamino)ethyl)chitins: Preparation and properties of novel aminated chitin derivatives. Macromolecules.

[34] Liu H., Yang Q., Zhang L., Zhuo R., Jiang X. (2016). Synthesis of carboxymethyl chitin in aqueous solution and its thermo- and pH-sensitive behaviors. Carbohydr. Polym..

[35] Jayakumar R., Prabaharan M., Nair S.V., Tokura S., Tamura H., Selvamurugan N. (2010). Novel carboxymethyl derivatives of chitin and chitosan materials and their biomedical applications. Prog. Mater. Sci..

[36] Chandrasekaran M., Kim K.D., Chun S.C. (2020). Antibacterial Activity of Chitosan Nanoparticles: A Review. Processes.

[37] El-Azab H., Remiz Y., Radwan M., Sadek M. (2020). Synthesis and Characterization of Chitosan based Catalyst for Catalysis Applications. Int. J. Adv. Trends Comput. Sci. Eng..

[38] Safari J., Azizi F., Sadeghi M. (2015). Chitosan nanoparticles as a green and renewable catalyst in the synthesis of 1,4-dihydropyridine under solvent-free conditions. New J. Chem..

[39] Pedroso-Santana S., Fleitas-Salazar N. (2020). Ionotropic gelation method in the synthesis of nanoparticles/microparticles for biomedical purposes. Polym. Int..

[40] Hoang N.H., Le Thanh T., Sangpueak R., Treekoon J., Saengchan C., Thepbandit W., Papathoti N.K., Kamkaew A., Buensanteai N. (2022). Chitosan Nanoparticles-Based Ionic Gelation Method: A Promising Candidate for Plant Disease Management. Polymers.

[41] Bonev B., Hooper J., Parisot J. (2008). Principles of assessing bacterial susceptibility to antibiotics using the agar diffusion method. J. Antimicrob. Chemother..

[42] Yang Y., Cai Z., Huang Z., Tang X., Zhang X. (2018). Antimicrobial cationic polymers: From structural design to functional control. Polym. J..

[43] Gauba A., Rahman K.M. (2023). Evaluation of Antibiotic Resistance Mechanisms in Gram-Negative Bacteria. Antibiotics.

[44] Kritchenkov A.S., Lipkan N.A., Kurliuk A.V., Shakola T.V., Egorov A.R., Volkova O.V., Meledina T.V., Suchkova E.P., Zabodalova L.A., Dysin A.P. (2020). Synthesis and Antibacterial Activity of Chitin Tetrazole Derivatives. Pharm. Chem. J..

[45] Dupont H., Montravers P., Zak O., Sande M.A. (1999). Chapter 21—Rat Polymicrobial Peritonitis Infection Model. Handbook of Animal Models of Infection.

[46] Shakola T.V., Rubanik V.V., Kurliuk A.V., Kirichuk A.A., Tskhovrebov A.G., Egorov A.R., Kritchenkov A.S. (2023). Benzothiazole Derivatives of Chitosan and Their Derived Nanoparticles: Synthesis and In Vitro and In Vivo Antibacterial Effects. Polymers.

[47] Egorov A.R., Kirichuk A.A., Rubanik V.V., Tskhovrebov A.G., Kritchenkov A.S. (2023). Chitosan and Its Derivatives: Preparation and Antibacterial Properties. Materials.

[48] Nathan C., Cunningham-Bussel A. (2013). Beyond oxidative stress: An immunologist’s guide to reactive oxygen species. Nat. Rev. Immunol..

[49] Uttara B., Singh A.V., Zamboni P., Mahajan R.T. (2009). Oxidative stress and neurodegenerative diseases: A review of upstream and downstream antioxidant therapeutic options. Curr. Neuropharmacol..

[50] Forman H.J., Zhang H. (2021). Targeting oxidative stress in disease: Promise and limitations of antioxidant therapy. Nat. Rev. Drug Discov..

[51] Kongkaoroptham P., Piroonpan T., Pasanphan W. (2021). Chitosan nanoparticles based on their derivatives as antioxidant and antibacterial additives for active bioplastic packaging. Carbohydr. Polym..

[52] Neha K., Haider M.R., Pathak A., Yar M.S. (2019). Medicinal prospects of antioxidants: A review. Eur. J. Med. Chem..

[53] Natera J., Massad W., García N.A. (2012). The role of vitamin B6 as an antioxidant in the presence of vitamin B2-photogenerated reactive oxygen species. A kinetic and mechanistic study. Photochem. Photobiol. Sci..

[54] Matxain J.M., Ristilä M., Strid Å., Eriksson L.A. (2006). Theoretical study of the antioxidant properties of pyridoxine. J. Phys. Chem. A.

[55] Ilyasov I.R., Beloborodov V.L., Selivanova I.A., Terekhov R.P. (2020). ABTS/PP decolorization assay of antioxidant capacity reaction pathways. Int. J. Mol. Sci..

